# CircCOL1A2 Sponges MiR-1286 to Promote Cell Invasion and Migration of Gastric Cancer by Elevating Expression of USP10 to Downregulate RFC2 Ubiquitination Level

**DOI:** 10.4014/jmb.2112.12044

**Published:** 2022-05-11

**Authors:** Hang Li, Lixin Chai, Zujun Ding, Huabo He

**Affiliations:** Gastroenterology and Hepatobiliary Surgery, The Affiliated Hospital of Hangzhou Normal University, Hangzhou 310015, Zhejiang, P.R.China

**Keywords:** Gastric cancer, circCOL1A2, miR-1286, USP10, RFC2

## Abstract

Gastric cancers (GC) are generally malignant tumors, occurring with high incidence and threatening public health around the world. Circular RNAs (circRNAs) play crucial roles in modulating various cancers, including GC. However, the functions of circRNAs and their regulatory mechanism in colorectal cancer (CRC) remain largely unknown. This study focuses on both the role of circCOL1A2 in CRC progression as well as its downstream molecular mechanism. Quantitative polymerase chain reaction (qPCR) and western blot were adopted for gene expression analysis. Functional experiments were performed to study the biological functions. Fluorescence in situ hybridization (FISH) and subcellular fraction assays were employed to detect the subcellular distribution. Luciferase reporter, RNA-binding protein immunoprecipitation (RIP), co-immunoprecipitation (Co-IP), RNA pull-down, and immunofluorescence (IF) and immunoprecipitation (IP) assays were used to explore the underlying mechanisms. Our results found circCOL1A2 to be not only upregulated in GC cells, but that it also propels the migration and invasion of GC cells. CircCOL1A2 functions as a competing endogenous RNA (ceRNA) by sequestering microRNA-1286 (miR-1286) to modulate ubiquitin-specific peptidase 10 (USP10), which in turn spurs the migration and invasion of GC cells by regulating RFC2. In sum, CircCOL1A2 sponges miR-1286 to promote cell invasion and migration of GC by elevating the expression of USP10 to downregulate the level of RFC2 ubiquitination. Our study offers a potential novel target for the early diagnosis and treatment of GC.

## Introduction

Gastric cancers (GC) develop as malignant tumors in the digestive tract and are reported to be the fifth most common carcinoma and the third-leading cause of cancer-associated mortality across the globe [1, 2]. Risk factors for the incidence of GC include helicobacter pylori infection, tobacco intake, age, sex, and diets high in salty and smoked foods [3]. Endoscopic resection and perioperative chemotherapy have been developed to treat GC according to the stage. Combination chemotherapy, such as platinum salt plus fluoropyrimidine, has been regarded as the standard of care [4]. Despite the progress made in treatment options for GC, the mortality rate and prognosis remain unsatisfactory due to the difficulty in timely diagnosis [5]. Most early-stage GC is asymptomatic or features only mild symptoms. Moreover, most GC patients are diagnosed at the advanced stage after the cancer cells have already metastasized to seriously threaten their life and health [2]. Therefore, it is important to study the underlying mechanisms of GC cell migration and invasion.

Circular RNAs (circRNAs), characterized by their covalently closed structure, are associated with the progression of multiple cancers [6]. A large number of reports concerning the roles of circRNAs in GC have emerged. For instance, circFAM73A facilitates the cancer stem cell-like properties of GC via regulating the miR-490-3p/HMGA2 axis and β-catenin stabilization mediated by HNRNPK [7]; circLMO7 sponges miR-30a-3p to propel GC progression through the WNT2/β-catenin pathway [8]; and circFGD4 has been found to attenuate GC cell progression through regulation of the miR-532-3p/APC axis [9]. Exploring the roles of circRNAs in the incidence and development of GC is crucial to finding potential targets for the early diagnosis and effective treatment of GC patients. As the circRNA of our study, circCOL1A2 has been reported to propel the migratory and invasive abilities of tumor cells in tongue squamous cell carcinoma [10]. Its host gene, collagen type I alpha 2 (COL1A2), is recognized as a new biomarker for GC and promotes GC cell progression by targeting the PI3k-Akt signaling pathway [11, 12]. However, the effects of circCOL1A2 on GC cell migration and invasion have never before been studied.

High-throughput sequencing technology has been widely used for cancer study and in the analyses of differentially expressed genes and cancer biomarkers [13]. ‘Bioinformatics’ refers to the utilization of mathematical, statistical and computational approaches to process and analyze biological data; it can be used to profile cancer-specific genes [14]. In our study, we utilized high-throughput sequencing technology and bioinformatics to screen out circCOL1A2 before performing experiments to probe into its role and mechanisms. Finally, by researching the regulatory mechanism underlying migration and invasion of gastric cancer, we are able to offer insight into novel, targeted therapies for this disease.

## Materials and Methods

### Cell Line Culture

GES-1, AGS, HGC-27, MKN-45 and 293T cells were commercially acquired from ATCC (USA). All the cells were cultured in DMEM (MD207-050, Gibco-BRL/Invitrogen, USA), along with 10% FBS (16000-044, Gibco, USA) and 1% Penicillin-Streptomycin Solution under the condition of 5% CO_2_ at 37°C.

### Vector Construction

Full-length sequences of RFC2 were inserted into pcDNA3.1 vector for the construction of overexpression vectors, with vector itself as negative control (NC). Short hairpin RNA (shRNA) targeting circCOL1A2 and USP10 as well as sh-NC were constructed for interference. The overexpression and knockdown of miR-1286 were conducted using miR-1286 mimics and inhibitor.

### Quantitative Polymerase Chain Reaction (qPCR)

qPCR was carried out as described before [15]. The total RNAs were extracted from GES-1, AGS, HGC-27 and MKN-45 cells using TRIzol reagent (T9108, Takara, Japan). After the removal of genomic DNA, the RNAs were subjected to reverse-transcription by Hifair III 1st Strand cDNA Synthesis SuperMix (11141ES10, Takara) for qPCR. The samples were measured using a qRT-PCR kit (QR0100-1KT, Sigma-Aldrich, USA). The results were calculated on a basis of 2^-ΔΔCt^. Bio-repeats were implemented in triplicate.

### Western Blot

Western blot was carried out as described previously [16]. The total proteins of AGS, MKN-45 and 293T cells were extracted by using RIPA lysis buffer (KeyGEN BioTECH, China). Afterwards, the total proteins were treated with 10% SDS/PAGE Resolving Gel Master Mix (P0670-250ml, Beyotime, China) for separation, followed by transfer onto polyvinylidene fluoride (PVDF) membranes (T2234, Thermo Fisher, USA). Then, 5% skim milk was adopted to block the membranes, which were then subjected to incubation with primary antibodies overnight at 4°C. After the washes, the membranes were incubated with secondary antibodies for 1 h at room temperature. The results were then visualized and recorded. β-Actin was used as an internal reference. Bio-repeats were implemented in triplicate. The primary antibodies used in this assay include Anti-β-actin, Anti-MMP2 (ab181286, Abcam, UK), Anti-MMP9 (ab228402, Abcam), Anti-USP10 (ab109219, Abcam) and Anti-RFC2 (ab174271, Abcam).

### Transwell Assay

Transwell assay was implemented as described previously [16]. The transwell assay, with or without Matrigel, was designed to assess GC cell invasion and migration respectively. The transfected cells were put into the upper chamber with serum-free medium, with complete medium supplemented to the lower chamber. After 24 h, the cells in the upper chamber were abraded. Following the fixation, the cells in the lower chambers were stained by 0.2% crystal violet solution before counting under the microscope. Bio-repeats were implemented in triplicate.

### Wound Healing Assay

Wound healing assay was conducted as described previously [16]. GC cells were prepared in the 6-well plates. The wounds were scraped by pipette tips, followed by washing. The wound widths were examined at 0 h and 24 h by Image J.

### Fluorescence In Situ Hybridization (FISH) and Subcellular Fraction Assays

These two assays were implemented as reported previously [17]. FISH was performed using the RiboTM Fluorescent In Situ Hybridization Kit (RiboBio, China). GC cells were treated with 4% PFA for fixation. We then used 0.5% Triton X-100 to permeabilize the cells for 15 min at 4°C. Subsequently, digoxigenin-labeled (DIG-labeled) target gene probe or control probe mix was used to incubate the cells for 4 h at 55°C. Hoechst-conjugated Anti-DIG antibodies were adopted to detect signals. A laser confocal microscope was employed for image obtaining. DAPI (D9542, Sigma-Aldrich) was used to counterstain nuclei. Bio-repeats were implemented in triplicate.

A Nuclear and Cytoplasmic Extraction Reagent Kit (Thermo Scientific) was employed to perform the subcellular fractionation, followed by measurement of the extracted RNAs by qPCR. U6 or β-actin was utilized as the nuclear or cytoplasmic control.

### RNA-Binding Protein Immunoprecipitation (RIP) Assay

RIP assay was carried out as reported previously [17]. Cells were lysed with RIPA lysis buffer. Cell lysate was subjected to incubation with magnetic beads conjugated with Anti-Ago2 (ab186733, Abcam) and Anti-IgG (ab6789, Abcam). The precipitated RNAs were measured by qPCR. Bio-repeats were implemented in triplicate.

### RNA Pull-Down Assay

RNA pull-down assay was carried out as reported previously [17]. The structure buffer was added into 1 μg of biotin-labeled circCOL1A2 or USP10. Then, biotinylated circCOL1A2/USP10 was heated and ice-bathed for 3 min for denaturing. Following that, 15 μl of streptavidin beads were added into biotin-labeled and denatured RNA for a 2h-incubation at 4°C. The magnetic bead-probe complex was then mixed with the cell lysate and incubated overnight at 4°C. After incubation, the RNA was extracted by Trizol and measured by qPCR for miR-1286 enrichment. Bio-repeats were implemented in triplicate.

### Dual-Luciferase Reporter Assay

Dual-luciferase reporter assay was carried out as reported previously [17]. For this assay, pmirGLO-circCOL1A2/USP10-Wt was created by subcloning the sequences of circCOL1A2/USP10 into pmirGLO vectors. Likewise, circCOL1A2/USP10 with mutated binding sites to miR-1286 seed region was subcloned into vectors for the creation of pmirGLO-circCOL1A2/USP10-Mut. Next, after culturing 293T cells in 96-well plates, we transfected them with the reporter vectors and mimics of miR-1286, or NC mimics. The empty vectors themselves were used as NC. The relative activity of luciferase was analyzed. Renilla Luciferase was used as the internal reference. Bio-repeats were implemented in triplicate.

### Co-Immunoprecipitation (Co-IP) Assay

Co-IP assay was carried out as reported previously [18]. Flag-RFC2 was constructed for 48 h-transfection with 293T cells, which were then lysed using RIPA lysis buffer, followed by the addition of Anti-Flag-RFC2 and Anti-USP10, as well as Anti-IgG. Then, A/G beads were employed to capture the complexes containing the proteins and antibodies. After the elution in PBS, the bound proteins were denatured for western blot. Bio-repeats were implemented in triplicate.

### Immunocytofluorescence (IF) Assay

IF assay was carried out as described previously [18]. GC cells were inoculated in 96-well plates. Subsequently, the cells were fixed by 4% formaldehyde for 20 min, followed by permeabilization using 0.1% Triton X-100. Next, the cell samples were incubated with Anti-USP10 and Anti-RFC2 at 4°C overnight, followed by incubation with the secondary antibodies at room temperature for 1 h. DAPI staining was adopted to counterstain the cell nuclei. A fluorescence microscope (XSP-63B, Shanghai Optical Instrument Factory, China) was utilized to observe immunofluorescence. Bio-repeats were implemented in triplicate.

### Immunoprecipitation-Western Blot (IP-Western Blot) Assay

IP assay was carried out as reported previously [19]. Flag-RFC2 was constructed, followed by transfection into 293T cells for 48 h. The 293T cells were then lysed and incubated with Anti-Flag-RFC2 overnight at 4°C. Protein A/G-beads were added to the complex for 4 h-cultivation at 4°C. Lastly, the precipitated proteins were transferred to SDS-PAGE gel for western blot.

### Stability Assays

These assays were conducted as reported previously [20]. GC cells were cultured to detect RNA and protein stability. To assess the stability of circCOL1A2 and COL1A2, RNase R (RNR07250, Epicentre, USA) was used to treat the cells. After treatment with RNase R, qPCR was used to compare the expressions of circCOL1A2 and COL1A2. For analysis of protein stability, western blot was used to detect the level of RFC2 at 0, 3, 6, 9 h, respectively, after treatment with 50 μm cycloheximide (CHX). Bio-repeats were implemented in triplicate.

### Statistical Analysis

Student’s *t*-test and one-way/two-way analysis of variance (ANOVA) were adopted to compare differences between groups. A *p*-value of under 0.05 was considered to indicate statistical significance.

## Results

### CircCOL1A2 with a Circular Structure Is Upregulated in GC Tissues

**Fig. 1:**
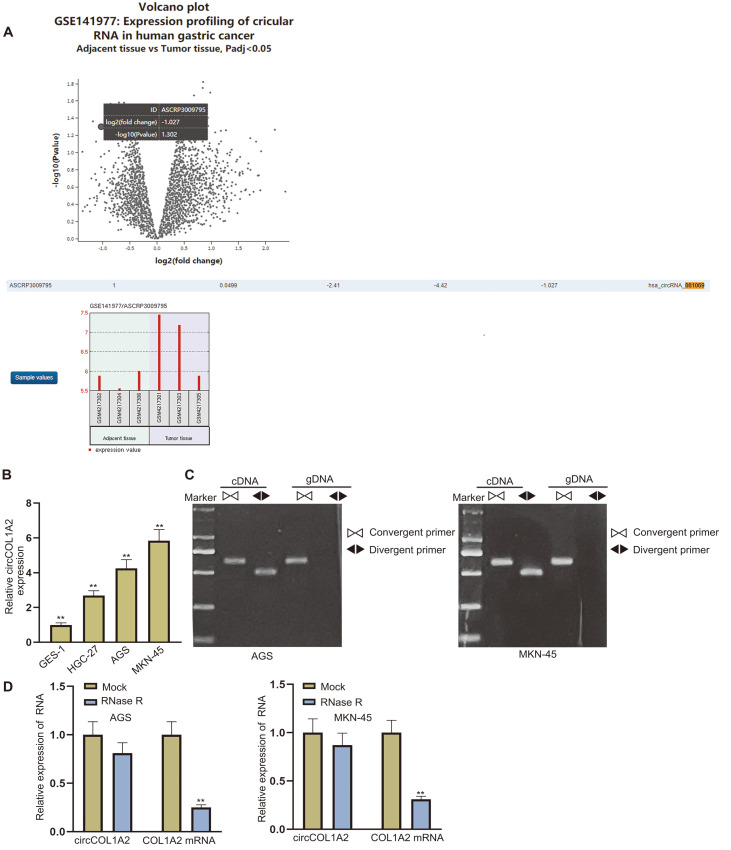
CircCOL1A2 with a circular structure is upregulated in GC tissues. (**A**) GeoChip was utilized to screen out differentially expressed circRNAs in GC. (**B**) The expression of circCOL1A2 was detected by qPCR in HGC-27, AGS, MKN-45 and GSE-1 cells. (**C**) PCR-agarose gel electrophoresis detected the circular structure of circCOL1A2 in AGS and MKN-45 cells. (**D**) CircCOL1A2 and COL1A2 mRNA were detected by qPCR in RNase R-treated AGS and MKN-45 cells to assess the stability. One-way ANOVA and two-way ANOVA were used for comparison detection. **p < 0.01.

We utilized GeoChip to analyze circRNAs differentially overexpressed in GC tissues. We identified circRNA_105040 and circRNA_081069 based on GSE141977, which includes 3 GC tumors and 3 adjacent non-tumor tissues (Fig. 1A). CircRNA_105040, which has the highest fold change, has been studied in GC previously [21]. Therefore, we selected circRNA_081069, with the second highest fold change, for our study. CircRNA_081069 was termed as circCOL1A2, as its host gene is COL1A2. Subsequently, qPCR was used to detect the expression of circCOL1A2 in GC cell lines (HGC-27, AGS and MKN-45) and human gastric mucosa epithelial cell line (GSE-1). As a result, circCOL1A2 was found to be upregulated in the GC cell lines (Fig. 1B). Due to their higher expressions of circCOL1A2, AGS and MKN-45 cells were selected for follow-up experiments. Next, PCR-agarose gel electrophoresis showed that circCOL1A2 was amplified in complementary DNA (cDNA) by both convergent primer and divergent primer, while it could not be amplified in genomic DNA (gDNA) by divergent primer, which verified the circular structure of circCOL1A2 (Fig. 1C). We then performed qPCR to detect the expressions of circCOL1A2 and COL1A2 mRNA after the treatment with RNase R. The expression of circCOL1A2 was revealed as remaining almost unchanged, while that of COL1A2 mRNA was significantly reduced (Fig. 1D). As circRNAs feature relatively stable structures, we further validated the circular structure of circCOL1A2. To sum up, circCOL1A2 with a circular structure is upregulated in GC tissues.

### CircCOL1A2 Promotes Cell Migration and Invasion of GC

**Fig. 2:**
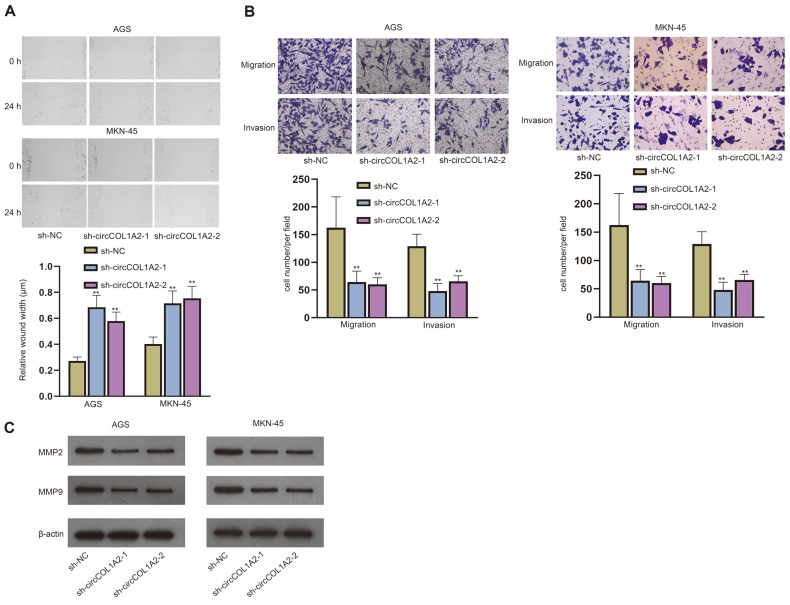
CircCOL1A2 promotes cell migration and invasion of GC. (**A**) Wound healing assay detected GC cell migration after circCOL1A2 depletion. (**B**) Transwell assay in GC cells tested cell migration and invasion after circCOL1A2 depletion. (**C**) Western blot assessed the levels of MMP2 and MMP9 after circCOL1A2 depletion. One-way ANOVA was used for comparison detection. **p < 0.01.

Next, we detected the effects of circCOL1A2 on GC cell progression. First, qPCR was used to detect the expression of circCOL1A2 after the transfection of sh-circCOL1A2-1/2/3, and showed that the plasmids inhibited the expression of circCOL1A2 (Fig. S1A). Due to the higher efficiency, sh-circCOL1A2-1 and sh-circCOL1A2-2 were used for follow-up experiments. Afterwards, migration and invasion of AGS and MKN-45 cells were evaluated by wound healing and transwell assays after the knockdown of circCOL1A2. The results showed that circCOL1A2 ablation inhibited the migratory and invasive capacities of GC cells, as evidenced by the increased wound width and decreased cell number (Figs. 2A and 2B). MMP2 and MMP9 are migration marker proteins [22]. Hence, we used western blot to detect the effects of circCOL1A2 on MMP2 and MMP9 levels in AGS and MKN-45 cells. After the interference with circCOL1A2, the levels of MMP2 and MMP9 were decreased, indicating the suppression of cell migration (Fig. 2C). Taken together, circCOL1A2 promotes cell migration and invasion of GC.

### CircCOL1A2 Interacts with miR-1286 in GC Cells

**Fig. 3:**
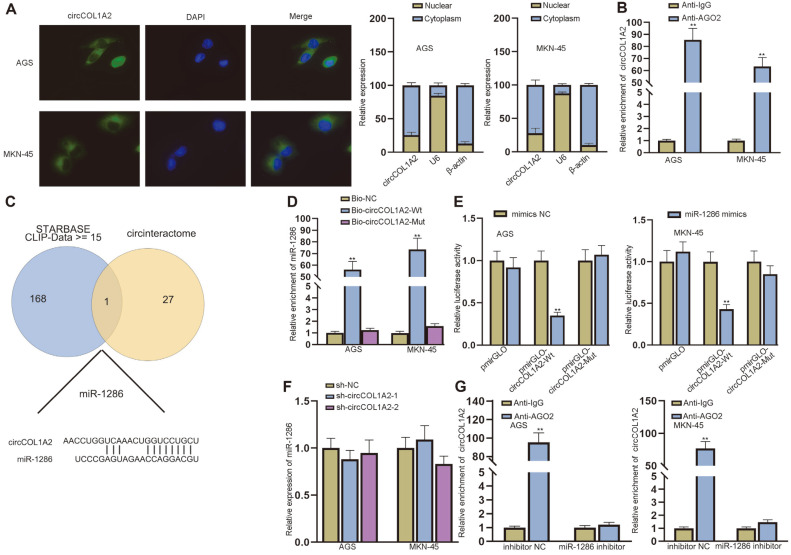
CircCOL1A2 interacts with miR-1286 in GC cells. (**A**) FISH and subcellular fraction assays detected the subcellular location of circCOL1A2 in AGS and MKN-45 cells. (**B**) RIP detected the enrichment of circCOL1A2 in Anti-AGO2 group. (**C**) The target miRNAs of circCOL1A2 in GC cells were predicted by starBase (http://starbase.sysu.edu.cn/) and Circular RNA Interactome (https://circinteractome.nia.nih.gov/). (**D-E**) RNA pull-down and luciferase reporter assays were implemented to detect the interaction between circCOL1A2 and miR-1286 in GC cells. (**F**) The expression of miR-1286 in GC cells was detected by qPCR after the ablation of circCOL1A2. (**G**) RIP detected the enrichment of circCOL1A2 in Anti-AGO2 group after the ablation of miR-1286. Student’s t-test, one-way ANOVA and two-way ANOVA were used for comparison detection. **p < 0.01.

We proved that circCOL1A2 can promote the migration and invasion of GC cells. As circCOL1A2 cannot encode proteins, we next probed into the underlying mechanisms of circCOL1A2 in GC cells. FISH and subcellular fraction assays were used to detect the subcellular location of circCOL1A2 in AGS and MKN-45 cells. The results showed that circCOL1A2 was located both in the nuclei and cytoplasm of GC cells, but mainly in the cytoplasm (Fig. 3A). Based on the subcellular location of circCOL1A2, we conjectured that circCOL1A2 might regulate GC cells via ceRNA mode. For verification, we conducted the RIP assay and found the enrichment of circCOL1A2 in the Anti-AGO2 group, indicating the existence of circCOL1A2 in RNA-induced silencing complex (RISC). The results proved the ceRNA mode of circCOL1A2 (Fig. 3B). Next, we utilized bioinformatics to screen the downstream target of circCOL1A2 (Fig. 3C). We identified miR-1286 based on the prediction of starBase (http://starbase.sysu.edu.cn/) and Circular RNA Interactome (https://circinteractome.nia.nih.gov/). Subsequently, miR-1286 mimics and inhibitors led to the increase and reduction in miR-1286 expression respectively, as verified by qPCR (Figs. S1B and S1C). RNA pull-down and luciferase reporter assays were performed to detect the interaction between miR-1286 and circCOL1A2. According to the results, miR-1286 binds to circCOL1A2 in GC cells, as evidenced by the enrichment of miR-1286 in the Bio-circCOL1A2-Wt group, and decreased luciferase activity in the pmirGLO-circCOL1A2-Wt group (Figs. 3D and 3E). We then performed qPCR to detect miR-1286 expression after the ablation of circCOL1A2 and found that circCOL1A2 could not influence the expression of miR-1286 (Fig. 3F). However, the RIP assay using miR-1286 inhibitor showed that the enrichment of circCOL1A2 in Anti-AGO2 group was inhibited by miR-1286 ablation (Fig. 3G). The abovementioned results indicated that circCOL1A2 acts as a sponge of miR-1286.

### CircCOL1A2 Regulates USP10 by Competitively Binding to miR-1286

**Fig. 4:**
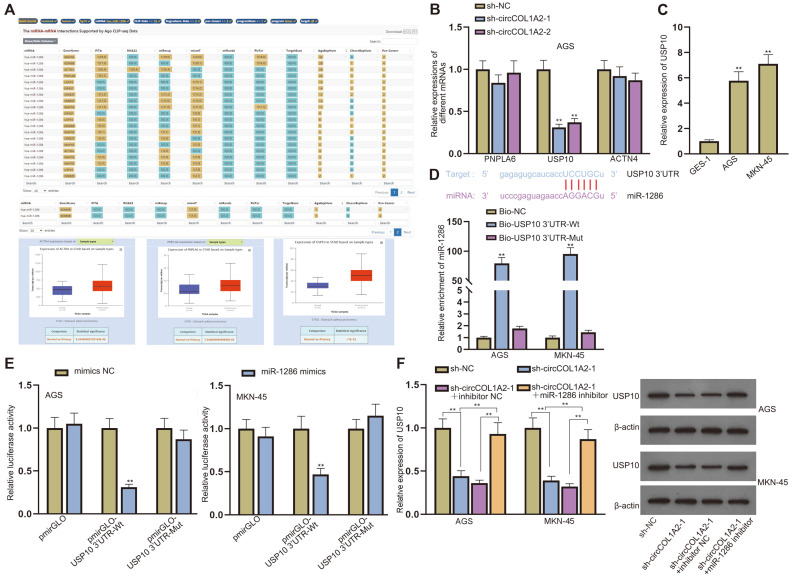
CircCOL1A2 regulates USP10 via competitively binding to miR-1286. (**A**) UALCAN (http://ualcan.path.uab.edu/) and starBase were utilized to predict the target mRNAs of miR-1286. (**B**) The expressions of PNPLA6, USP10 and ACTN4 were detected by qPCR in AGS cells after the knockdown of circCOL1A2. (**C**) USP10 expression in AGS, MKN-45 and GES-1 cells was detected by qPCR. (**D-E**) The binding sites between USP10 3’UTR and miR-1286 were shown. The interaction between USP10 and miR-1286 in GC cells was proved by RNA pull-down and luciferase reporter assays. (**F**) Western blot and qPCR were implemented to detect USP10 mRNA and protein levels in GC cells transfected with sh-NC, sh-circCOL1A2-1, sh-circCOL1A2-1+inhibitor NC or sh-circCOL1A2-1+miR-1286 inhibitor. One-way ANOVA and two-way ANOVA were used for comparison detection. **p < 0.01.

As previous results proved that circCOL1A2 binds to miR-1286, we then explored the target mRNA of miR-1286. The downstream mRNAs of miR-1286 were predicted by starBase under certain conditions (CLIP data >=18; Degradome-Data >=2; pan-Cancer >=2). We next used UALCAN (http://ualcan.path.uab.edu/) to predict the expressions of candidate mRNAs in GC tumors. To ensure stringency, we only selected genes with AgoExpNum>=15. Five candidate genes were selected. KDM6B [23] and MAP1B [24] have been studied thoroughly and therefore lack research value. ACTN4 has been proved to be a novel therapeutic target for GC [25]. There is a dearth of reliable research reports on phospholipase PNPLA6. The role of deubiquitinase USP10 in GC has been investigated in a previous study [26], which was consistent with our expectations. In addition, USP10 is highly expressed in GC tumors according to the data from bioinformatics (Fig. 4A). For further validation, qPCR was performed to detect the expressions of PNPLA6, USP10 and ACTN4 after the knockdown of circCOL1A2 in AGS cells. The results showed that only USP10 expression decreased significantly (Fig. 4B). Afterwards, we detected USP10 expression in AGS, MKN-45 and GES-1 cells and found that USP10 was high-expressed in GC cells (Fig. 4C). Thus, we selected USP10 as the focus of our study. The binding sites between USP10 3’UTR and miR-1286 were predicted. RNA pull-down and luciferase reporter assays validated the interaction between miR-1286 and USP10 3’UTR (Figs. 4D and 4E). Rescue experiments were conducted to explore the relationships among circCOL1A2, miR-1286 and USP10. The qPCR and western blot results revealed detection of the mRNA and protein levels of USP10 after the transfection of sh-NC, sh-circCOL1A2-1, sh-circCOL1A2-1+inhibitor NC or sh-circCOL1A2-1+miR-1286 inhibitor. The miR-1286 inhibitor was found to counteract the inhibitory effect of circCOL1A ablation on USP10 at protein and mRNA levels (Fig. 4F). Taken together, circCOL1A2 regulates USP10 via competitively binding to miR-1286.

### USP10 Downregulates RFC2 Ubiquitination Level in GC Cells

**Fig. 5:**
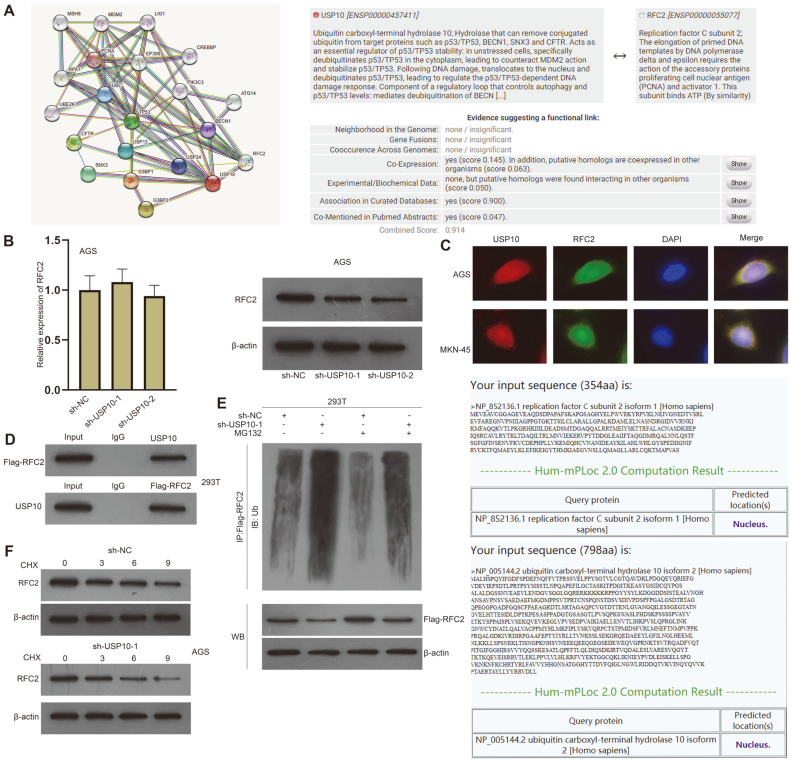
USP10 downregulates RFC2 ubiquitination level in GC cells. (**A**) STRING database (https://string-db.org/) and UALCAN were used to predict the proteins interacting with USP10 and RFC2 expression in GC. (**B**) Western blot and qPCR were used to evaluate RFC2 level after the ablation of USP10. (**C**) IF assay and bioinformatics proved the co-localization of USP10 and RFC2 in the nuclei of GC cells. (**D**) Co-IP in 293T cells assessed the interaction between RFC2 and USP10. (**E**) IP and western blot detected the ubiquitination level of RFC2 in 293T cells. (**F**) Western blot detected RFC2 level after CHX treatment and USP10 interference in AGS cells. One-way ANOVA was used for comparison detection.

It has been reported that USP10 can be used as a biomarker of GC [27], but its specific regulatory mechanisms in GC remain to be explored. Therefore, we probed into its role in GC. We first analyzed the proteins interacting with USP10. STRING database (https://string-db.org/) was used to predict the proteins binding to USP10. USP10, as an important deubiquitinase, interacts with a considerable number of proteins, but most of them are deubiquitinases of the same family and are key factors of other pathways. Comparatively, RFC2 has a high possibility of interacting with USP10 and belongs to the replication factor C family, which has been reported to promote cell invasion and migration of various cancers [28]. UALCAN was used to predict the expression of RFC2 in GC, showing its association with GC (Fig. 5A). We then conducted qPCR and found that RFC2 expression was upregulated after the transfection of pcDNA3.1-RFC2, and USP10 expression was depleted after the transfection of sh-USP10-1/2/3 (Figs. S1D and S1E). Because of higher efficiency, sh-USP10-1 and sh-USP10-2 were selected for assays. Western blot and qPCR were used to detect the mRNA and protein levels of RFC2 after interference with USP10. As a result, USP10 ablation was shown to inhibit the protein level of RFC2 in AGS cells, instead of mRNA level (Fig. 5B). The IF assay validated the co-localization of USP10 and RFC2 in the nuclei of GC cells. The prediction of Hum-mPLoc 2.0 (http://www.csbio.sjtu.edu.cn/bioinf/hum-multi-2/) also proved the same (Fig. 5C). The interaction between USP10 and RFC2 was proved by Co-IP assays in 293T cells (Fig. 5D). IP and western blot were used to detect the ubiquitination level of RFC2 in 293T cells. The results showed that the ubiquitination level of RFC2 was increased after interference with USP10, but was reversed by the addition of MG132 (Fig. 5E). As MG132 is a proteasome inhibitor, this indicates that USP10 affects RFC2 protein level through the ubiquitin-proteasome mechanism. Afterward, we performed A western blot to detect the degradation of RFC2 after CHX (a transcription inhibitor) treatment and USP10 interference. The results showed that in CHX-treated AGS cells, the degradation of RFC2 was accelerated after USP10 interference, which further verified that USP10 mediates the ubiquitination level of RFC2 to stabilize RFC2 protein (Fig. 5F). Taken together, USP10 downregulates the RFC2 ubiquitination level in GC cells.

### USP10 Promotes Migration and Invasion of GC Cells by Regulating RFC2 Expression

**Fig. 6:**
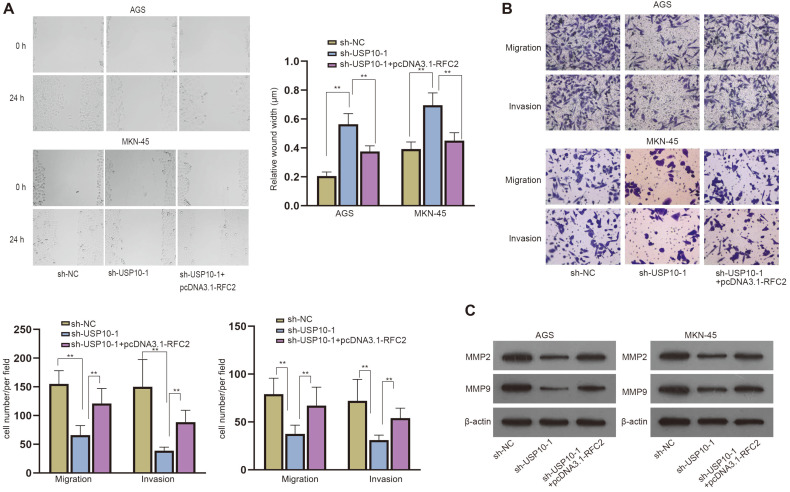
USP10 promotes migration and invasion of GC cells by regulating RFC2 expression. (A-B) Wound healing and transwell assays were implemented to evaluate cell migration and invasion of GC after the transfection with sh-NC, sh-USP10-1 or sh-USP10-1+pcDNA3.1-RFC2. (**C**) Western blot assessed the levels of MMP2 and MMP9 after the transfection with sh-NC, sh-USP10-1 or sh-USP10-1+pcDNA3.1-RFC2. One-way ANOVA was used for comparison detection. **p < 0.01.

Next, we used rescue experiments to verify that USP10 promotes GC cell progression by regulating RFC2. We performed wound healing and transwell assays in GC cells transfected with sh-NC, sh-USP10-1 or sh-USP10-1+pcDNA3.1-RFC2. The results showed that RFC2 enhancement reversed the increased wound width and decreased cell number by USP10 ablation (Figs. 6A and 6B). The western blot results showcased that MMP2 and MMP9 levels were reduced by USP10 ablation, but were then countervailed by RFC2 overexpression (Fig. 6C). In conclusion, USP10 promotes migration and invasion of GC cells by regulating RFC2 expression.

## Discussion

GC is one of the most common cancers and is characterized by high mortality, due to the difficulty of diagnosis and treatment. Advanced-stage GC patients suffer from the risk of metastasis. Therefore, it is vitally important to study the mechanisms underlying GC cell migration and invasion. Various studies have proven that circRNAs participate in the regulation of GC, including circFAM73A [7], circLMO7 [8] and circFGD4 [9].

We identified circCOL1A2 using the GEO database. CircCOL1A2 has been reported to facilitate the migratory and invasive capacities of cancer cells in tongue squamous cell carcinoma [10]. However, the effects of circCOL1A2 on cell migration and invasion in GC have never been reported. We verified the circular structure of circCOL1A2 using electrophoresis and stability analysis. Moreover, qPCR showed that circCOL1A2 was overexpressed in GC cells, indicating their interrelationship. Wound healing, transwell, and western blot assays proved that circCOL1A2 facilitates cell migration and invasion of GC. Furthermore, FISH results showed that circCOL1A2 was mainly located in the cytoplasm of GC cells, suggesting the ceRNA mode. We then utilized the database to screen potential miRNAs. Next, luciferase reporter, RIP and RNA pull-down assays were conducted to ensure that miR-1286 was the downstream target of circCOL1A2.

USP10 was identified as the potential target of miR-1286 using bioinformatics and qPCR. Again we employed the luciferase reporter, RIP and RNA pull-down assays to verify that miR-1286 interacts with USP10. USP10 has been reported to promote cell proliferation of hepatocellular carcinoma via deubiquitinating and stabilizing YAP/TAZ [29]; USP10 suppresses the growth and invasive capacity of lung cancer cells by overexpressing PTEN [30]; USP10 modulates oncogene-induced senescence through deubiquitination and stabilization of p14ARF [31]; USP10 propels cell proliferation in colon cancer through deubiquitinating and stabilizing Musashi-2 [32]. However, its role in GC cells has rarely been reported. We used bioinformatics, qPCR and western blot to screen out the protein interacting with USP10, namely, RFC2. RFC2 has been reported to be correlated with various malignancies. For instance, RFC2, targeted by miR-744, regulates the cell cycle and proliferation of colorectal cancer cells [33]. Additionally, RFC2 was found to modulate the cell cycle and DNA replication of liver cancer [34]. However, RFC2 ubiquitination has never been studied in GC. We performed gene expression analysis and IF assay and found that USP10 affects the protein level of RFC2 and is co-localized in the nucleus with RFC2. The results of Co-IP validated the interaction between RFC2 and USP10. IP and a western blot showed that USP10 downregulates RFC2 ubiquitination level in GC cells. The results of western blot using CHX showed that USP10 affects the stability of RFC2. Rescue experiments demonstrated that USP10 promotes migration and invasion of GC cells by regulating RFC2.

To sum up, our study demonstrated that circCOL1A2 sponges miR-1286 to promote cell invasion and migration of GC by elevating the expression of USP10 to downregulate RFC2 ubiquitination level. The present study is the first to prove that circCOL1A2 regulates USP10 via sequestering miR-1286, thereby promoting GC cell migration and invasion. In addition, we have verified for the first time that USP10 facilitates GC cell migration and invasion by regulating RFC2 expression. Exploring the mechanisms of circCOL1A2 in GC cells offers insight into therapeutic targets for GC diagnosis and treatment. That said, our study has room for improvement in some respects. For instance, no in vivo verification was conducted in this study. Nude mice can be adopted to assess the effect of circCOL1A2 on GC cells. Furthermore, the clinicopathological relevance of circCOL1A2 remains to be explored. In the future study, we will conduct in vivo experiments and clinicopathological analysis to further our understanding of circCOL1A2 in GC.

## Supplemental Materials

Supplementary data for this paper are available on-line only at http://jmb.or.kr.
