# Supplementary material for: CircCOL1A2 Sponges MiR-1286 to Promote Cell Invasion and Migration of Gastric Cancer by Elevating Expression of USP10 to Downregulate RFC2 Ubiquitination Level
Source: J Microbiol Biotechnol. 2022 May 11;32(7):938–48. doi: 10.4014/jmb.2112.12044 (PMC9628928; doi:10.4014/jmb.2112.12044)
Supplement: Supplementary file 1 [file jmb-32-7-938-supple.pdf]

J. Microbiol. Biotechnol.
https://doi.org/10.4014/jmb.2112.12044

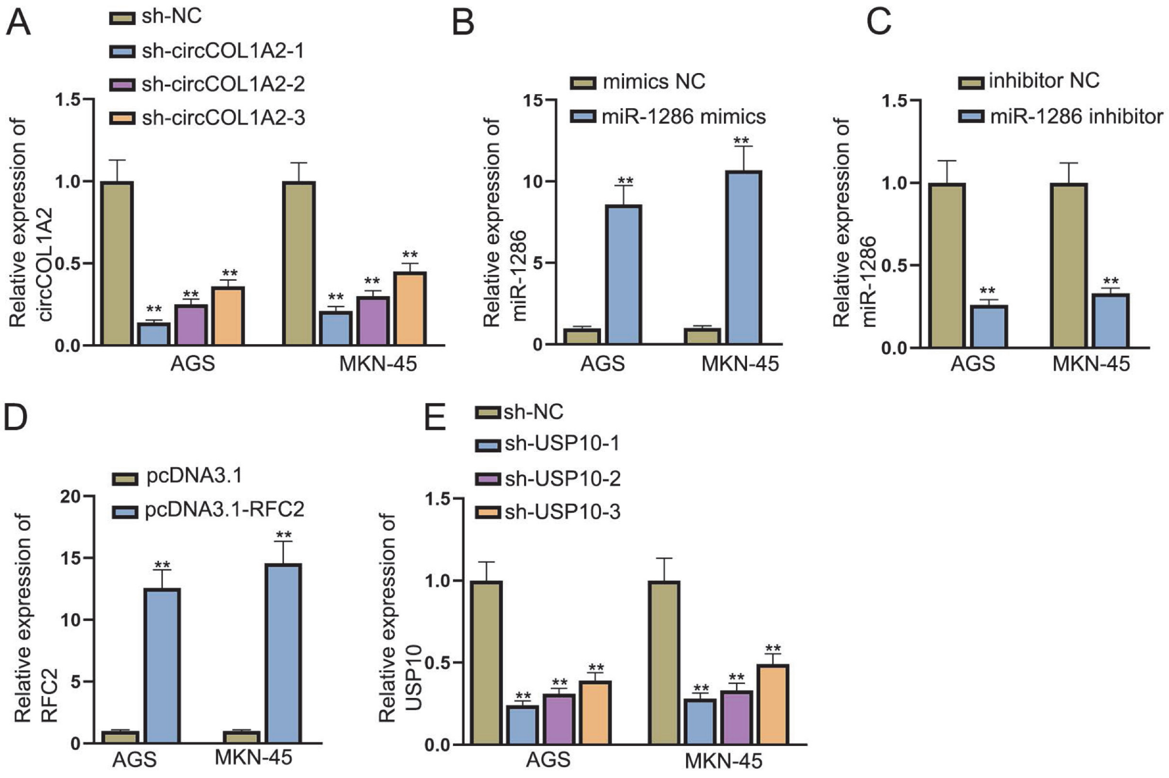

Fig. S1. The efficiencies of plasmids. (A-E) The efficiencies of sh-circCOL1A2-1/2/3, miR-1286 mimics, miR-1286 inhibitor, pcDNA3.1-RFC2 and sh-USP10-1/2/3 were assessed by qPCR. Student's *t*-test and one-way ANOVA were used for comparison detection. $^{**}p < 0.01$.
